# Another “great mimicker”: FDG-PET/CT imaging findings of sarcoid-like reaction

**DOI:** 10.1259/bjrcr.20150060

**Published:** 2015-07-03

**Authors:** A T K Kendi, B J Barron, D Bonta, R K Halkar, B Rathore, D M Schuster

**Affiliations:** ^1^ Division of Nuclear Medicine and Molecular Imaging, Department of Radiology and Imaging Sciences, Emory University School of Medicine, Atlanta, GA, USA; ^2^ Veterans Affairs Medical Center, Atlanta, GA, USA

## Abstract

Sarcoid-like reaction (SLR) is a cause of non-caseating granulomas in some of the cancer patients with otherwise no signs or symptoms of sarcoidosis. SLR has been described in a variety of solid organ malignancies, including breast and lung cancer. SLR may result in hypermetabolic activity in 18-fludeoxyglucose positron emission tomography (PET)/CT scan, resulting in false positive reporting for malignancy. The purpose of this case series is to expose residents/practising physicians who interpret PET/CT to a series of cases illustrating findings of SLR.

## Case review

### Case 1

The patient was a 63-year-old male with adenocarcinoma of the rectosigmoid junction and multifocal tubular adenoma of the right colon. The patient underwent right hemicolectomy and 6 cycles of folinic acid (also called leucovorin, FA or calcium folinate)/fluorouracil (5FU)J/oxaliplatin (FOLFOX).

**Figure 1.**
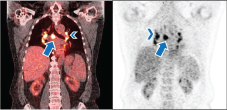
Case 1. A 63-year-old male post surgery and chemotherapy for colonic tumour. Post-therapy coronal fused PET/CT (a) and coronal PET (b) show mediastinal (arrow, a,b) and symmetric bilateral hilar hypermetabolic adenopathy (arrowhead, a,b). The biopsy result was consistent with sarcoid-like reaction. PET, positron emission tomography.

Findings: Post-therapy positron emission tomography (PET)/CT scan showed bilateral symmetric hilar and mediastinal enlarged lymph nodes with increased 18-fludeoxyglucose (FDG) activity (Figure 1). The maximum standardized uptake value (SUV max) of the subcarinal lymph node was 11.7, SUV max of the right hilar lymph nodes was 10.6 and that of the left hilar lymph nodes was 9. There were no lung abnormalities to suggest active infection or inflammation. Biopsy of one of these lymph nodes was consistent with sarcoid-like reaction (SLR).

### Case 2

The patient was a 45-year-old female with multifocal multicentric left breast cancer, mcT2N0M0, Stage 2A, oestrogen receptor/progesterone receptor+, human epidermal growth factor 2(−). The patient had chemotherapy followed by left mastectomy and reduction of the right breast. She completed radiation therapy afterwards and started chemotherapy.

**Figure 2.**
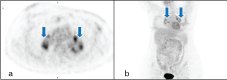
A 45-year-old female post therapy for breast cancer. Axial positron emission tomography image (a) and coronal maximum intensity projection image (b) show interval development of symmetric hilar adenopathy with intermediateJ 18-fludeoxyglucose activity (arrows).

**Figure 3.**
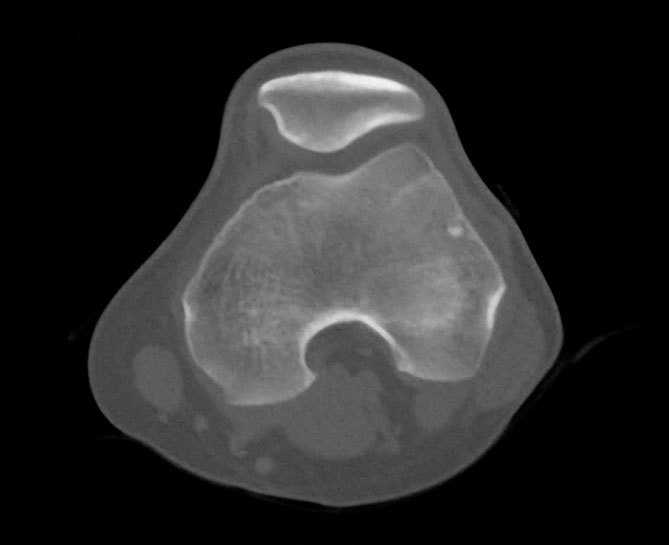
A 45-year-old female post therapy for breast cancer. Follow-up PET/CT, axial PET (a) and coronal maximum intensity projection (b) images show complete improvement of symmetric hilar lymph nodes. PET, positron emission tomography.

Findings: PET/CT scan after completion of radiation therapy revealed hypermetabolic symmetric hilar lymph nodes (Figure 2). SUV max of the right hilar lymph nodes was 8.6 and SUV max of the left hilar lymph nodes was 8.5. There were no lung findings to suggest active infection. Upon review of the history with the patient’s oncologist, this appearance was accepted as most consistent with SLR. Follow-up PET/CT scan revealed interval resolution of hypermetabolic lymph nodes (Figure 3).

### Case 3

A 57-year-old male with non-Hodgkin’s lymphoma presented initially with a left lower extremity swelling. The patient was found to have a large mass in the left iliac wing and was diagnosed with diffuse B-cell lymphoma. The patient underwent 6 cycles of rituximab and Cytoxan (cyclophosphamide)/Adriamycin (hydroxy doxorubicin)/vincristine (Oncovin)/prednisone (also known as CHOP).

**Figure 4.**
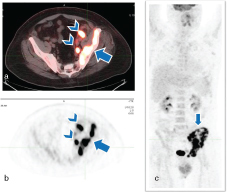
A 57-year-old male with non-Hodgkin’s lymphoma. Axial fused PET/CT (a), axial PET (b) and coronal maximum intensity projection (c) revealed a left iliac wing hypermetabolic mass (arrow) and left pelvic hypermetabolic adenopathy (arrowheads, a,b). PET, positron emission tomography.

**Figure 5.**
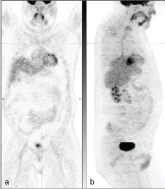
A 57-year-old male with non-Hodgkin’s lymphoma. First PET/CT following therapy: Coronal reformatted PET (a) and sagittal maximum intensity projection (b) show complete "metabolic" response to therapy. PET, positron emission tomography.

**Figure 6.**
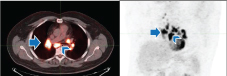
A 57-year-old male with non-Hodgkin’s lymphoma. Second follow-up PET/CT: Axial fused PET/CT (a) and coronal maximum intensity projection (b) images of the chest revealed new hypermetabolic mediastinal (arrowhead) and hilar adenopathy (arrows). Biopsy was consistent with sarcoid-like reaction. PET, positron emission tomography.

**Figure 7.**
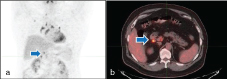
A 57-year-old male with non-Hodgkin’s lymphoma. The third follow-up PET/CT: Coronal maximum intensity projection (a) and axial fused PET/CT (b) show reduction in avidity of uptake of the mediastinal lymph nodes with interval development of mildly hypermetabolic abdominal lymph nodes (arrows). PET, positron emission tomography.

**Figure 8.**
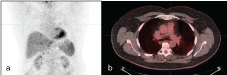
A 57-year-old male with non-Hodgkin’s lymphoma. Last follow-up PET/CT: Coronal maximum intensity projection (a), axial fused PET/CT (b) showing resolution of uptake in precaval and mediastinal lymph nodes. PET, positron emission tomography.

Findings: Initial PET/CT showed a lytic hypermetabolic mass in the left iliac wing with left pelvic hypermetabolic adenopathy (Figure 4). Biopsy of the bone lesion revealed diffuse B-cell lymphoma. The first post-treatment PET/CT scan performed 8 months after completion of therapy showed a complete "metabolic" response (Figure 5). The second follow-up PET/CT scan 2 years after completion of therapy demonstrated new mediastinal/hilar hypermetabolic adenopathy (Figure 6). SUV max of the precarinal lymphadenopathy was 18.7, subcarinal lymphadenopathy was 26.3 and that of the right hilar lymph nodes was 24.6. Biopsy of one of these lymph nodes revealed non-caseating granulomatous inflammation. The third follow-up PET/CT scan after 2 years showed new abdominal hypermetabolic adenopathy (Figure 7) with SUV max of 8.7. The last follow-up PET/CT performed 2 years after the third follow-up PET/CT scan showed almost complete improvement of these findings (Figure 8). The patient did not receive any therapy after the first follow-up PET/CT scan. The second to last follow-up PET/CTs were acquired to monitor SLR-related findings. There was spontaneous resolution of FDG activity of these nodes, thus it was not a treatment-induced tumour response.

## Discussion

Pathophysiology of SLR is not completely understood. There are, in general, two forms, localized (around the tumour) and general (distant sites from primary tumour). The localized form of SLR could be from degenerative/necrotic changes within the tumour.^1,2^ The general SLR might be from humoral or T-cell mediated factors, resulting in activation of macrophages.^1^

SLR is more common with patients undergoing restaging PET/CT scan rather than initial staging PET/CT scan.^1^

There are a variety of uptake patterns at PET/CT. The most common patterns are mediastinal nodal and/or symmetric hilar uptake. Other presentation patterns include pulmonary abnormalities, such as pulmonary nodules, ground-glass opacities, regional lymph nodes, liver, spleen or bone marrow uptake.^1–6^

## Learning points

1. SLR can be seen in a variety of solid-organ malignancies, either at the time of diagnosis or up to several years after.
2. Symmetric mediastinal/hilar FDG activity, unexpected FDG activity in a lymph node, bone or organ in cases with otherwise complete response to therapy should be interpreted carefully for the presence of SLR.
3. The FDG-PET/CT pattern can suggest SLR and should be reported as such.
4. Awareness of SLR will prevent unnecessary biopsy and therapy, but close follow-up is warranted.
